# A systematic review of play invitation signals in great apes: ontogenetic rather than phylogenetic differences structure variation in ludic communicative displays

**DOI:** 10.1007/s10071-026-02081-1

**Published:** 2026-07-31

**Authors:** Daniel López-Valdés, Diana Platas-Neri

**Affiliations:** 1 Centro de Estudios Antropológicos, Facultad de Ciencias Políticas y Sociales, Universidad Nacional Autónoma de México, CDMX, México; 2 Instituto de Investigaciones Antropológicas, Universidad Nacional Autónoma de México, CDMX, México

**Keywords:** Play invitation signals, Social play, Cognition, Great apes, Gestural communication

## Abstract

Play invitation signals mark interactions as non-threatening and playful, serving as a key mechanism for coordinating social play among great apes. Within the framework of gestural origins theories, particularly the contrast between genetic codification and ontogenetic ritualization, this systematic review—conducted following PRISMA guidelines—examined empirical evidence on play invitation signals in *Pan troglodytes*, *Pan paniscus*, *Gorilla gorilla*, *Pongo pygmaeus*, and *Pongo abelii*. Studies were included if they provided developmental categorization or detailed age information and an ethogram explicitly describing play invitations. A total of 29 studies met these criteria. Results revealed marked ontogenetic variation across species, with play invitation signals peaking during infancy and juvenility, declining in subadults, and showing a secondary rise among adult females. These findings highlight the importance of developmental trajectories for understanding cognitive and communicative flexibility in great apes. They suggest that the structure of interspecific variation in play invitation repertoires does not align consistently with phylogenetic relationships, supporting a model in which socio-developmental factors play a substantial role in shaping ludic communicative displays.

## Introduction

Play is generally defined as a sequence of actions performed flexibly and often derived from behaviours used in other contexts, temporarily detached from their primary functions (Burghardt 2005). It has also been proposed that play may constitute a distinct behavioural system, although the nature of such a system remains debated (Pellis et al. 2019). Play has been proposed to fulfil multiple functions, including training for unexpected challenges (training for the unexpected hypothesis, Špinka et al. 2001), self- and social assessment (Paquette 1994), anxiety reduction (Palagi et al. 2004, 2006, 2007; Norscia and Palagi 2011), conflict resolution, and the enhancement of social cohesion (Palagi 2023). Although play does not always require explicit signalling—for instance, in mother–infant interactions or in solitary play—social play between peers often relies on voluntary engagement and on communicative displays that signal the interaction as playful rather than aggressive or otherwise serious.

This need is especially evident in social play, particularly in play fighting, because players often recruit motor patterns that are also used in potentially risky contexts such as aggression. As a result, playful exchanges may be ambiguous and, unless properly regulated, may be misunderstood and even escalate into aggression (Pellis and Pellis 1996; Palagi & Cordoni, 2012; Cordoni et al. 2023). At the same time, play fighting does not simply reproduce aggression, as the two differ in important structural features (Smith 1997; Cordoni et al. 2023). Animals may therefore use specific communicative displays, including facial expressions, body postures, locomotor displays and vocalisations, to indicate the benign, non-serious nature of the interaction (Pellis and Pellis 1996; Palagi et al. 2016; Cordoni et al. 2023).

In the literature, communicative displays associated with playful interactions are broadly referred to as play signals (Pellis and Pellis 1996, 2017; Palagi et al. 2016). Within this broader category, play invitation signals constitute a more specific subset: signals used to initiate play by recruiting a potential partner into the interaction and establishing a shared play frame (Palagi 2008; Pellis and Pellis 1996, 2017). Depending on the species and context, these signals may include gestures, body postures, locomotor displays, facial expressions and vocalisations. In what follows, we use the term play invitation repertoire to refer to the set of distinct signal types reported in the literature as used to initiate play within a given species or developmental stage.

Comparative studies in great apes and other mammals have revealed both shared patterns and species-specific differences in play invitation signals. Their expression may vary as a function of social context, including partner relationship quality, play intensity and developmental stage (Fröhlich et al. 2016; Palagi et al. 2004; Fröhlich and van Schaik 2022); the direction of these effects is not uniform across species or contexts, however, and relationship quality should be understood as a factor that modulates signal use rather than as a defining property of play invitation signals. Altogether, these findings indicate that the expression of play invitation signals is shaped jointly by social and developmental factors, raising the question of how this variation relates to broader debates on the origins of gestural communication, a question we turn to next.

Play invitation signals can be expressed through different communicative modalities, including gestures, body postures, locomotor displays, facial expressions and vocalisations. Facial expressions, in particular, are not unique to humans but represent a widespread communicative modality in mammals. Visible facial movements can convey socially relevant information to group members and appear to have evolved long before the emergence of modern humans (Waller and Micheletta 2013; Waller et al. 2020). Playful facial expressions, such as the play face documented across great apes and other mammals, are part of this broader evolutionary continuum. Among these modalities, gestures have received particular attention in studies of primate communication because they are often considered flexible, socially learned signals. The evolutionary origins of gestures in nonhuman primates have been widely debated, and several non-exclusive frameworks have been proposed (for reviews, see Liebal et al. 2019; Pika and Fröhlich 2019; van Boekholt et al. 2024): the social negotiation hypothesis (Pika and Fröhlich 2019), social transmission through imitation, the recently proposed recruitment hypothesis (Graham et al. 2025), and the two frameworks on which we focus here.

For the specific purpose of this review, we concentrate on the two accounts that most directly oppose each other regarding the role of an innate repertoire. The ritualization hypothesis (Halina et al. 2013; Tomasello et al. 1994) argues that gestures acquire communicative meaning through repeated social interactions and learning, whereas the genetic codification hypothesis (Genty and Byrne 2010; Hobaiter and Byrne 2017) posits that they are shaped by evolutionary selection, reflecting inherited species-specific repertoires. These two frameworks generate different expectations about the structure of variation in play invitation repertoires. Under a strong form of the genetic codification hypothesis, repertoires are expected to be shaped largely by inherited, species-typical predispositions; accordingly, the degree of overlap among species should be congruent with their phylogenetic relationships, such that more closely related taxa share more similar repertoires. Under the ritualization hypothesis, repertoires are instead expected to be shaped by repeated social interaction across development; overlap should therefore be structured by shared socio-developmental and contextual conditions and may cut across phylogenetic boundaries. We emphasise that both frameworks are compatible with developmental change in signal use—‘innate’ in this context does not imply signals that are present at birth, rigid, or immune to developmental effects (Byrne et al. 2017)—so the mere presence of ontogenetic variation does not adjudicate between them. Rather, our aim is to assess whether the *structure* of similarities and differences in play invitation repertoires aligns more closely with phylogenetic relationships or with developmental trajectories.

Play invitation signals provide an especially informative behavioural domain for evaluating these debates, for several reasons. First, prior work has shown that they are flexibly produced and strongly modulated by social context, partner identity, and play intensity (Fröhlich et al. 2016; Palagi et al. 2004; Fröhlich and van Schaik 2022); such context-sensitivity is more readily expected under accounts that emphasise repeated social interaction than under a strong form of genetic codification, which would predict more stereotyped use. Second, play invitations are often multimodal, combining gestures, postures, locomotor displays, facial expressions, and vocalisations; this combinatorial flexibility offers multiple dimensions along which variation across individuals, contexts, and species can be expressed. Third, play—and thus play invitation—occurs throughout the lifespan, from infancy to adulthood, which makes this domain particularly well suited to examining ontogenetic variation within a single signal category. These properties make play invitation signals a useful test bed for comparing developmental and phylogenetic sources of variation, while leaving open the empirical question of which source predominates. Because the genetic codification and ritualization hypotheses generate different predictions about how repertoire structure should be organised across species and across development, examining play invitation signals through this lens speaks directly to debates on the evolutionary and developmental origins of gestural communication in great apes, the question this review is designed to address.

In this review, we focus specifically on play invitation signals and, more precisely, on the composition and structural characteristics of their repertoires across species and developmental stages.

We do not examine signal frequency or communicative effectiveness in the present analysis. This exclusion does not imply that these dimensions are unimportant; rather, they are more strongly influenced by social opportunities, interaction rates, partner dynamics, and contextual variation. As a result, they are less suitable for evaluating broad comparative patterns across species and developmental stages.

In this study, we use the term ontogenetic differences to refer to variation in the composition of play invitation repertoires across developmental stages, that is, differences in the presence or absence of specific signal types during infancy, juvenility, subadulthood and adulthood. In contrast, phylogenetic differences refer to interspecific variation in repertoire composition and signal form across great ape taxa, while taking into account broad contextual factors such as study setting. This distinction allows us to systematically compare developmental and evolutionary sources of variation in ludic communicative displays.

Our objectives are twofold. First, using a phylogenetic approach, we characterise play invitation signals to identify interspecific patterns of variation in repertoire composition. Second, we analyse developmental trajectories within species to describe ontogenetic variation. By contrasting these dimensions, and following the predictions outlined above, we evaluate whether the structure of cross-species similarity tracks phylogeny or, instead, developmental and contextual factors.

Together, these considerations position play invitation signals as a particularly suitable behavioural domain for assessing developmental plasticity in relation to evolutionary constraints.

## Materials and methods

### Search strategy

We performed a systematic review of empirical primatological studies addressing play invitation signals in great apes, following the PRISMA 2021 guidelines (Page et al. 2021) to ensure transparency and replicability. Searches were performed in Web of Science, Scopus, and EBSCOhost between September and November 2023. The search was conducted in English, using the following query:

(“social play”) AND (“play invite” OR “communication” OR “gesture” OR “play face”) AND (“non-human primates” OR “great apes” OR “chimpanzees” OR “bonobos” OR “gorillas” OR “orangutans” OR “orang-utans”).

Only peer-reviewed empirical studies were considered. Records were screened by title, abstract, and full text. Duplicates, reviews, and studies without behavioral data were excluded.

Study selection and data extraction were performed by two reviewers independently. Discrepancies were resolved through discussion and, when necessary, by consulting a third author. We did not perform a formal risk-of-bias assessment, but we extracted information on study setting, sample size, and observational design as proxies for data quality and further replication and validity evaluation of our drawn conclusions.

### Inclusion criteria

(i) Studies performed in at least one of the following species: *Pan troglodytes*, *Pan paniscus*, *Gorilla gorilla*, *Pongo pygmaeus*, and *Pongo abelii*, (ii) that included play invitation signals in social play interactions, (iii) that individuals from the study group were categorized in the developmental stages or that their ages were available, and (iv) that they included an ethogram with the description of the play invitation signals. We included findings ranging from 1967, when the systematic study of play began (Fagen 1981; Loizos 1967), to November 2023. While our study focuses on great apes in general, other gorilla or orangutan species (e.g. *Gorilla beringei*, *Pongo tapanuliensis*) were left out due to no studies included during the initial search.

### Selection and synthesis

**Fig. 1:**
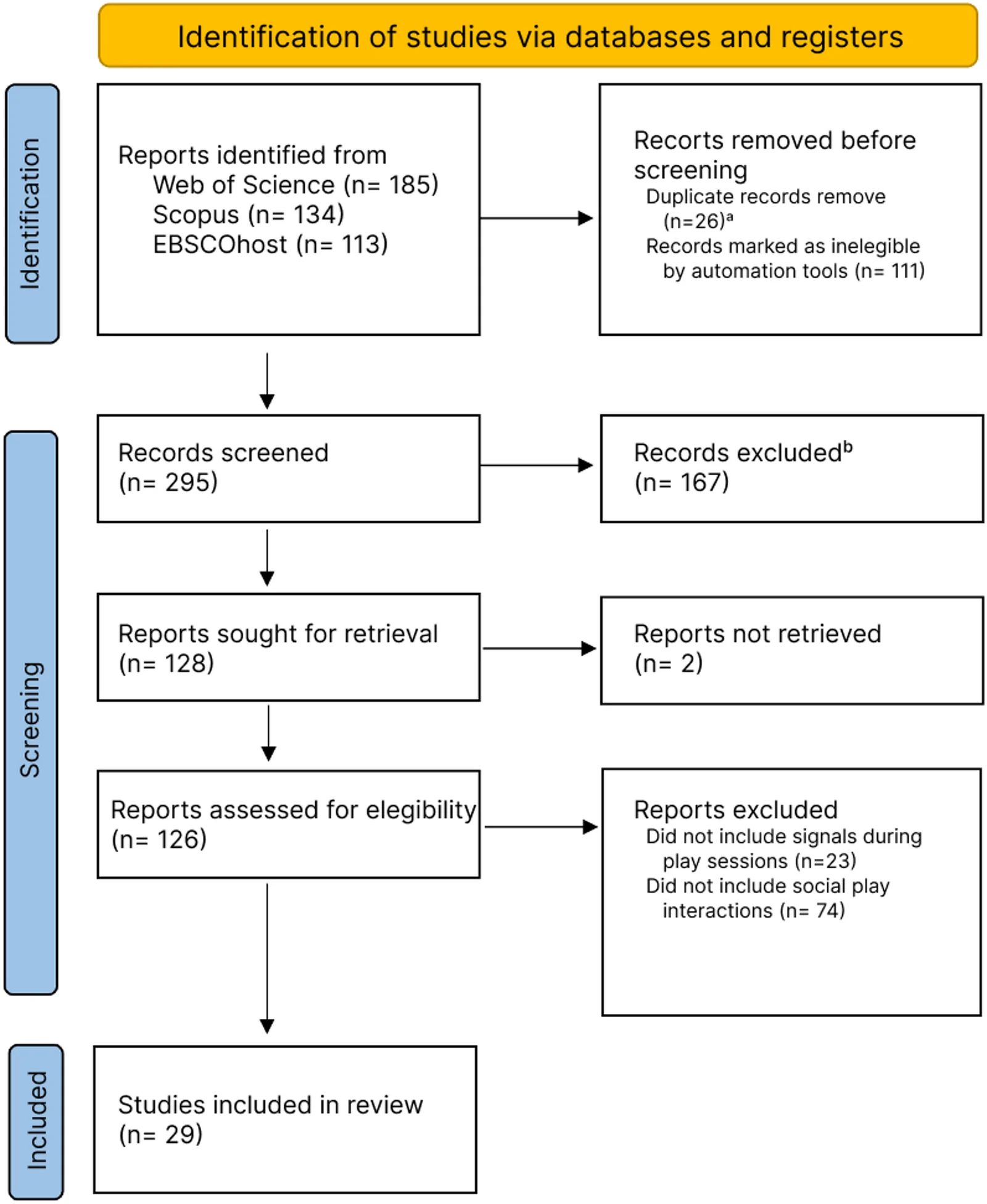
PRISMA 2020 flowchart for systematic reviews. Modified from de Page et al. (2021). Notes: a= Other reviews or non-empirical findings were excluded. b= Studies that reported findings in other species rather than our focus of interest.

After applying the inclusion criteria (Fig. 1), 29 studies were retained: 16 on *P. troglodytes*, 4 on *P. paniscus*, 4 on *G. gorilla*, 1 on *P. abelii*, and 4 comparative studies that included more than one species (e.g., combinations of *P. troglodytes*, *P. paniscus*, *P. pygmaeus*, and *P. abelii*).

### Data retrieval

We extracted data that matched the inclusion criteria, including studies reporting information from the same study group across different time points (e.g., Fröhlich et al. 2016, 2017; Roberts et al. 2014; Roberts and Roberts 2018). For each study, we recorded the following variables in a standardized spreadsheet: author(s), publication year, study title, species, group composition (number of individuals and age categories), study setting (captive or free-ranging), behaviors associated with play invitations, main results, and additional notes. When information was unclear or missing, we made no assumptions or imputations.

### Study subjects

The final dataset comprised 812 observed individuals in total: 564 *Pan troglodytes*, 59 *P. paniscus*, 51 *Gorilla gorilla*, 54 *Pongo pygmaeus*, and 84 *P. abelii* individuals. Of these, we identified at least 452 unique subjects: 53 of them were reported for more than one study, and information for 307 individuals did not report enough data for them to be identified as members absent from other samples (see Table 1 for details on the overlapping samples). Each individual was classified into one of four developmental stages—infant, juvenile, subadult, or adult—according to age definitions available in previous ethological and developmental literature (Table 2). Classification criteria were standardized across species to ensure comparability.

**Table 1:** Composition of the study groups within each retrieved study

Study	Year	*N*	Infants	Juveniles	Subadults	Adults	Setting
*Pan troglodytes*							
Amici & Liebal	2022	19	3	4	4	8	Captive
Bard et al.	2014	17	17	0	0	0	Captive
Cordoni & Palagi	2012	15	8	7	0	0	Captive
Davila-Ross et al.	2011	59	11	25	8	15	Free-ranging
Flack et al.^a^	2004	16	0	8	0	8	Captive
Fröhlich et al.^b^	2016	49	16	18	0	15	Free-ranging
Fröhlich et al.^b^	2017	49	16	18	0	15	Free-ranging
Gibson et al.^a^	2023	75	11	18	13	33	Free-ranging
Hobaiter & Byrne^a^	2011	81	18	15	16	32	Free-ranging
McCarthy et al.	2013	19	1	5	3	10	Captive
Mielke & Carvalho	2022	13	1	1	1	10	Free-ranging
Oliveira & Wasterlain	2020	15	0	4	0	11	Captive
Roberts et al.	2014	12	0	0	0	12	Free-ranging
Roberts et al.	2018	12	0	0	0	12	Free-ranging
Ross et al.	2014	7	7	0	0	0	Captive
Schneider et al.	2012	8	4	0	0	4	Captive
Spijkerman et al.^a^	1996	90	41	26	23	0	Captive
Tomasello et al.	1994	8	0	8	0	0	Captive
Total		564	150	157	68	185	
*Pan paniscus*							
Demuru et al.^b^	2015	12	4	1	0	7	Captive
Genty et al.	2014	10	0	0	7	3	Captive
Heesen et al.^a^	2022	14	0	3	4	7	Free-ranging
Palagi^b^	2008	11	2	1	0	8	Captive
Schneider et al.	2012	12	6	0	0	6	Captive
Total		59	12	5	11	31	
*Gorilla gorilla*							
Genty & Byrne	2009	30	6	2	9	13	Captive
Salmi & Muñoz	2020	9	2	1	0	6	Free-ranging
Waller & Cherry	2012	10	0	2	1	7	Captive
Weigel & Berman	2018	2	1	1	0	0	Captive
Total		51	9	6	10	26	
*Pongo pygmaeus*							
Cartmill & Byrne	2010	20	4	5	0	11	Captive
Fröhlich et al.	2022	13	0	4	3	6	Captive
	2022	21	0	8	3	10	Free-ranging
Total		54	4	17	6	27	
*Pongo abelii*							
Amici & Liebal	2022	16	2	2	6	6	Captive
Cartmill & Byrne	2010	8	3	1	0	4	Captive
Gruber	2014	11	4	7	0	0	Captive
Fröhlich & van Schaik	2022	18	0	5	5	8	Captive
Fröhlich & van Schaik^a^	2022	31	0	5	6	20	Free-ranging
Total		84	9	20	17	38	

a: Studies for which no full unique identifier was available to count individuals as unique study subjects. Either no individualised data was provided or only initial letters (e.g. “Ad”) were shown

b: Studies that shared the same sample across them (Fröhlich et al. 2016, 2017; and 4 members of Palagi 2008 were also present in Demuru et al. 2015)

**Table 2:** Developmental stages and its ages (in years) for each species

Species	Infants	Juveniles	Subadults	Adults	Sources
*Pan troglodytes*	0-4.9	5.0-8.9	9.0-12.9 (females) 9.0-15.9 (males)	≥ 13.0 (females) ≥ 16.0 (males)	Nishida et al. 2003
*Pan paniscus*	0-3.4	3.5–6.9	7.0-10.5	≥ 10.6	Boughner and Dean 2008
*Gorilla gorilla*	0-3.4	3.5–5.9	6.0-7.9	≥ 8.0	Kalpers et al. 2003; Robbins et al. 2009; Williamson and Gerald-Steklis 2001
*Pongo pygmaeus*	0-3.9	4.0-7.9	8.0-9.9 (females) 8.0-14.9 (males)	≥ 10.0 (females) ≥ 15.0 (males)	Kaplan and Rogers 2002; Kuze et al. 2005
*Pongo abelii*	0-6.9	7.0-9.9	10.0-14.9 (females) 10.0-13.9 (males)	≥ 15.0 (females) ≥ 14.0 (males)	Wich et al. 2004

### Phylogenetic comparison

#### Data extraction

A total of 456 play invitation signals were extracted from the ethograms reported in the included studies. Signals that shared equivalent definitions or descriptions were merged to avoid duplication, yielding a final dataset of 398 unique signals. Each signal was coded according to the categories described in Table 3, which specifies the criterion, its definition, variable type, and possible values.

The variables selected for the analysis capture structural dimensions of play invitation signals previously used in the literature on cross-species comparisons for primate gestural communication. Postural category and modality reflect the morphological and sensory properties of signals; these dimensions have been used to characterise overlap or differences – for instance, to explore the different degrees of social tolerance among chimpanzees or gorillas depending on the usage of tactile versus non-proximal ones (Cordoni et al. 2018; Hobaiter and Byrne 2011). Multimodality was included because the simultaneous usage of more than one sensory channel has been tied to emerging complexities during signal exchange (Liebal et al. 2006; Genty et al. 2009), while object use was included as a proxy for communicative elaboration beyond the signaller’s own body (Gibson et al. 2023). Sequentiality, understood as the execution of signals in combination directed at the same recipient, was included given its value as a proxy for communicative complexity and the construction of structured displays (Genty et al. 2009; McCarthy et al. 2013; Mielke and Carvalho 2022). The study setting (captive vs. free-ranging) was used as a control variable to account for differences in signal opportunities and social environments that could shape repertoires (Fröhlich and van Schaik 2022).

Vocalisations were not included in the present analysis. Although vocal signals form part of the broader play signal repertoire in great apes, vocal play invitations are inconsistently documented across the source studies, which differed substantially in whether and how vocal channels were coded alongside gestural and postural ones. To preserve comparability across studies and avoid systematic bias toward studies with detailed acoustic coding, we restricted the analysis to non-vocal signals. The role of vocal signals in play invitation merits dedicated systematic review.

#### Analysis

We conducted a Multiple Correspondence Analysis (MCA) to examine phylogenetic differences and to explore associations among signal characteristics, identifying latent patterns that could inform cross-species inferences. This method is a multivariate exploratory technique that identifies dimensions of covariation among multiple categorical variables, allowing to visualize structural relationships among observations, in this case, play signals (for another use case in primates see: Partan 2002). Prior to the analysis, the variable “Signal” was excluded due to its lack of discriminant variability and used only as the identifier of each entry within the dataset.

Dimensions explaining at least 10% of the total variance were retained and interpreted in detail. All analyses were conducted in RStudio (RStudio Team 2023) using the FactoMineR package v. 2.9 (Lê et al. 2008).

Following the MCA, we computed Mahalanobis distances between species level centroids in the two dimensional ordination space, which was defined by dimensions 1 and 2, these were responsible for the highest variation in the play invitation signals repertoires. Each centroid represented the mean coordinate of all signals attributed to a given species across the retrieved literature. The Mahalanobis distance rescales the separation between centroids by the pooled within-species covariance, so that it accounts for the dispersion of signals around each centroid rather than treating all directions in the ordination space as equivalent. Therefore, a larger distance indicates that the two compared species differ more in their characteristics of signals, as it was obtained by the MCA. Distance-to-centroid measures of this kind have been used in previous studies of primate communication to quantify dissimilarity among species-level signal distributions (Clink et al. 2017; Debracque et al. 2023).

**Table 3:** Analysis levels in the characterization of play invitation signals. Elaborated from Amici and Liebal 2022; Fröhlich and van Schaik 2022; Genty et al. 2009; Hobaiter and Byrne 2011; Liebal et al. 2006; Roberts et al. 2012, 2013; Roberts and Roberts 2019

Analysis level	Definition	Type of variable	Possible values
*General information*			
Signal	Name of the signal defined in the ethogram	Nominal	Name of the signal (See supplementary material)
Definition	Detailed description of the signals included in the ethogram of each species	Nominal	Definitions for each play invitation signal
Species	The species in which the signal was described	Categorical	Pt Pp Gg Py Pa
Postural category	See Table 4 for definitions	Nominal	Bodily movement Body postures Head movements and facial expressions Leg gestures Locomotory gait Manual
Modality	See Table 5	Nominal	Tactile Visual Auditory
Multimodality	Indicates whether the signal belongs to more than one of the described categories in Table 5	Binary (presence = 1, absence = 0)	1 0
Ontogenetic development observation	Presence of the signal across age and sex categories.	Binary (presence = 1, absence = 0)	Infants M/F; Juveniles M/F; Subadults M/F; Adults M/F.
*Play pattern*			
Sequentiality	Indicates whether the signal occurs in sequence with another one.	Binary (reported = 1, not reported = 0)	1 0
Use of objects	Describes whether the signal uses additional physical objects	Binary (presence = 1, absence = 0)	1 0
*Context setting*			
Captivity	Indicates whether the signal has been observed in captive groups	Binary (presence = 1, absence = 0	1 0
Free-ranging	Indicates whether the signal has been observed in free-ranging groups	Binary (presence = 1, absence = 0	1 0

Pt = Pan troglodytes; Pp = P. paniscus; Gg = Gorilla gorilla; Py = Pongo pygmaeus; Pa = Pongo abelii

**Table 4:** Postural categories identified for each signal. Elaborated from Roberts et al. 2012, 2013; Roberts and Roberts 2019

Category	Definition	Examples
*Bodily movement*	Non-locomotory whole-body movements that do not involve displacement through space, distinguished from locomotory gait.	Bow; Pirouette
*Body postures*	Static behavioral states adopted by the sender, in which the individual remains motionless.	Lay down; Belly-offer
*Head movements and facial expressions*	Movements of the head as a whole, as well as facial movements involving the mouth, lips, or other facial musculature.	Play face; Protruded lips
*Leg gestures*	Refers to signals conveyed through the lower limbs, including the feet.	Kick; Leg offer
*Locomotory gait*	Describes signals involving patterns of running or walking that convey intentional movement through space.	Tandem walk; Stiff walk
*Manual*	Signals executed with the upper limbs, including the hands.	Grab; Punch

**Table 5:** Modalities of signals. Elaborated with information from Hobaiter and Byrne 2011; Liebal et al. 2006; McCarthy et al. 2013; Roberts and Roberts 2019

Modality	Definition	Examples
Tactile	Signals involving physical contact with the recipient.	Tip head
Visual	Movements or postures that are perceptible to the recipient through sight, without involving physical contact and without generating auditory cues, regardless of the distance between sender and recipient.	Play face; Bow
Auditory	Signals generating sounds through non-vocal means (e.g., body or object contact). Vocalisations are not analysed in the present review (see Methods).	Water splash
*Multimodal*	Belonging to more than one of the above-mentioned modalities. In these cases, the predominant modality is assigned.	Clap (visual and auditory)

Signals belonging to a single modality (tactile, visual, or auditory) are referred to as unimodal

### Ontogenetic comparisons

#### Data extraction

To evaluate developmental differences across species, we extracted data on the presence of each signal type across ontogenetic stages. As in the phylogenetic comparison, each signal was coded according to the life stage(s) in which it was observed (see Table 3).

#### Analysis

For each species, we quantified the relative contribution of each ontogenetic stage to the total signal repertoire by dividing the number of signals recorded at that stage by the species’ total number of identified signals. Sex-specific percentages were then computed, and cross-species comparisons were performed to examine ontogenetic patterns and emerging interspecific trends.

## Results

### Phylogenetic comparison

Two dimensions accounted for at least 10% of the observed variation in play invitation signals, together explaining 33.40% of the cumulative variance.

**Fig. 2:**
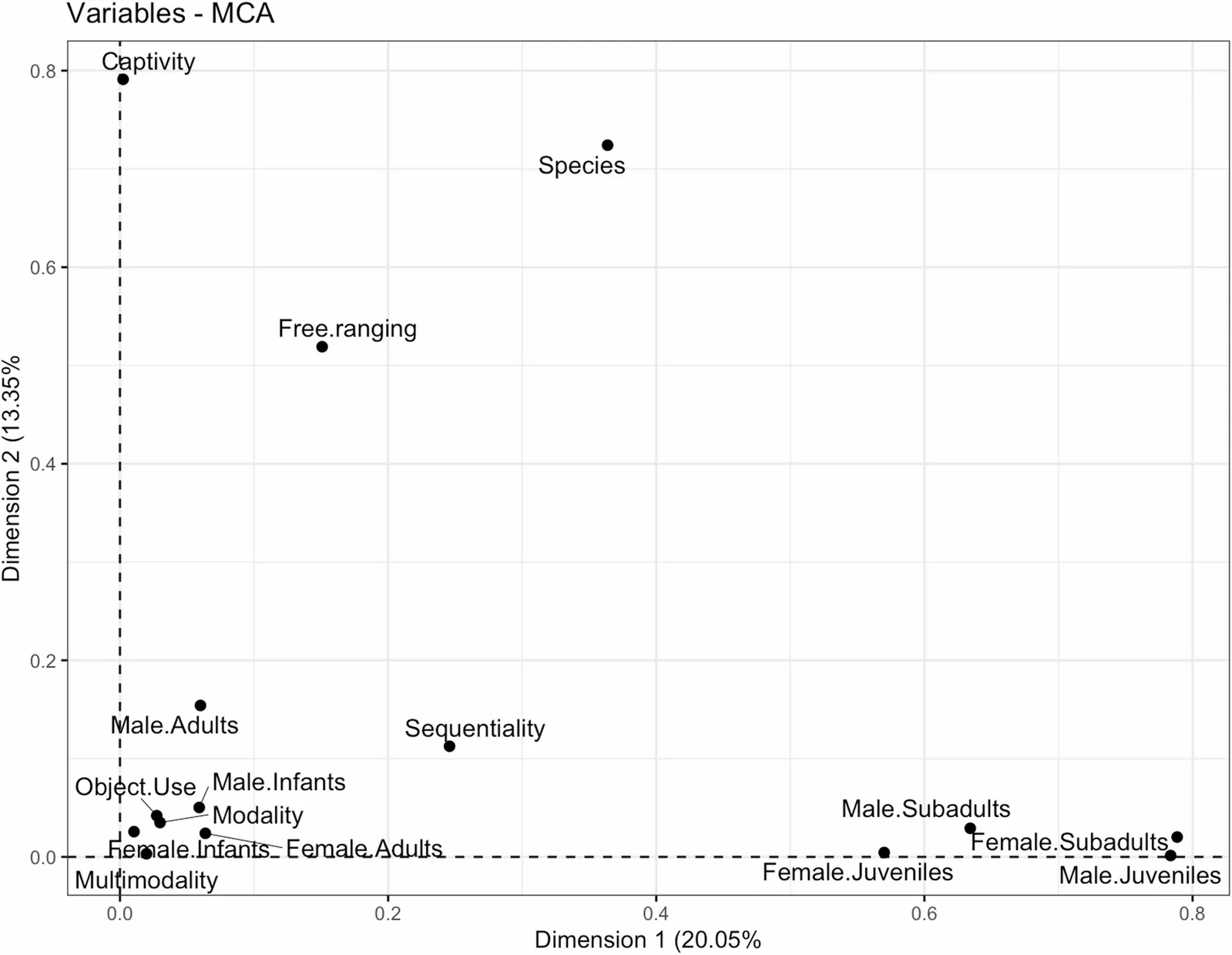
Variables related to the dimensions responsible for at least 10% of variance

The first dimension explained 20.05% of the variation and was primarily associated with ontogenetic stage and sex-related differences in signal use. Species identity also showed a moderate association with this dimension, greater than that of sequentiality, though smaller than its association with Dimension 2 (Fig. 2). Within this dimension, play signals associated with juvenile and subadult stages were most distinctly positioned from those associated with infants and adults, indicating that the primary axis of co-variation in signal characteristics corresponded to developmental stage rather than to species identity.

The second dimension explained 13.35% of the variation and was mainly related to study context (captive vs. free-ranging groups) and interspecific contrasts among species. Within dimension 2, captive-documented signals and free-ranging-documented signals loaded in opposing directions, with species-level groupings distributing along this axis in a pattern partially congruent with study context.

**Fig. 3:**
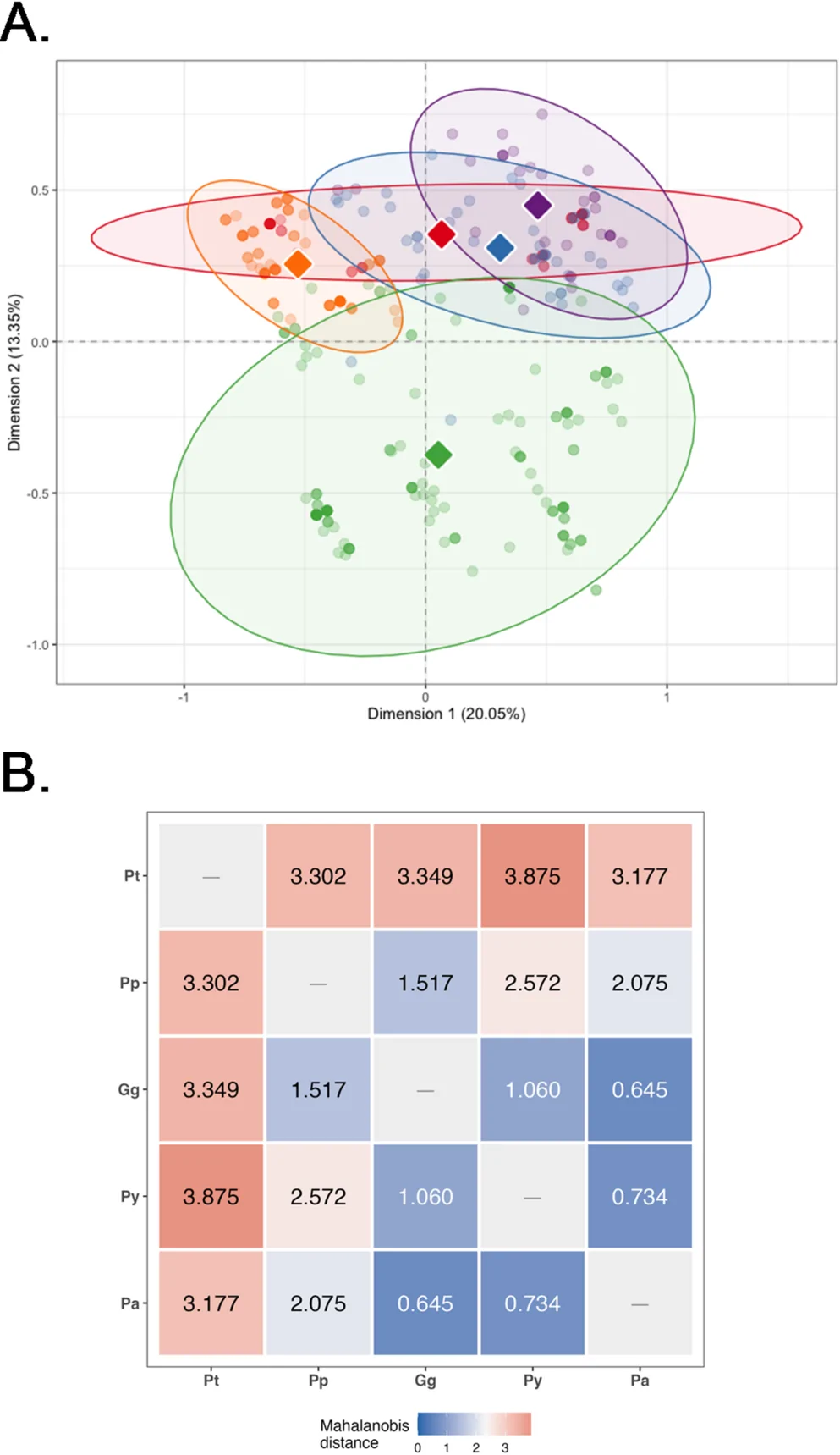
Phylogenetic comparison of play invitation signals in the great apes. **(A)** Scatterplot presenting play invitation signals across dimensions highlighting variance. Each point represents an individual signal; diamonds represent the species centroids; ellipses indicate the species groupings: *Pan troglodytes* (green), *Pan paniscus* (orange), *Gorilla gorilla* (red), *Pongo pygmaeus* (purple), and *Pongo abelii* (blue). **(B)** Pairwise species centroid distances in the Multiple Correspondence ordination space. Blue tones highlight proximity in species repertoires, red ones stand for differences between species

Across taxa, species groupings showed partial overlap in the ordination space (Fig. 3A). Chimpanzees exhibited the most diffuse distribution, spanning the lower portion of the plot and partially overlapping with all other taxa. The bonobo repertoire occupied a compact, distinct position in the upper left quadrant. The two *Pongo* species clustered together, with their invitation signals overlapping substantially, and gorillas occupied an intermediate position overlapping with both orangutan species.

After quantifying the spatial relationships between species-level centroids (Fig. 3B) we identified the shortest inter-species distance between *Gorilla gorilla* and *Pongo abelii* (0.645), followed closely by the two *Pongo* species (0.734), indicating close structural proximity in signal repertoire. The greatest distance was observed between *Pan troglodytes* and Bornean orangutans (3.875).

Notably, the distance between *Pan troglodytes* and *Pan paniscus* was among the largest in the matrix (3.302), despite their close phylogenetic relationship, while *Gorilla gorilla* was closer to both Pongo species (Gg–Py = 1.060; Gg–Pa = 0.645) than to either Pan species (Gg–Pt = 3.349; Gg–Pp = 1.517). These centroid distances do not consistently track the phylogenetic relationships among taxa.

### Ontogenetic comparisons

**Fig. 4:**
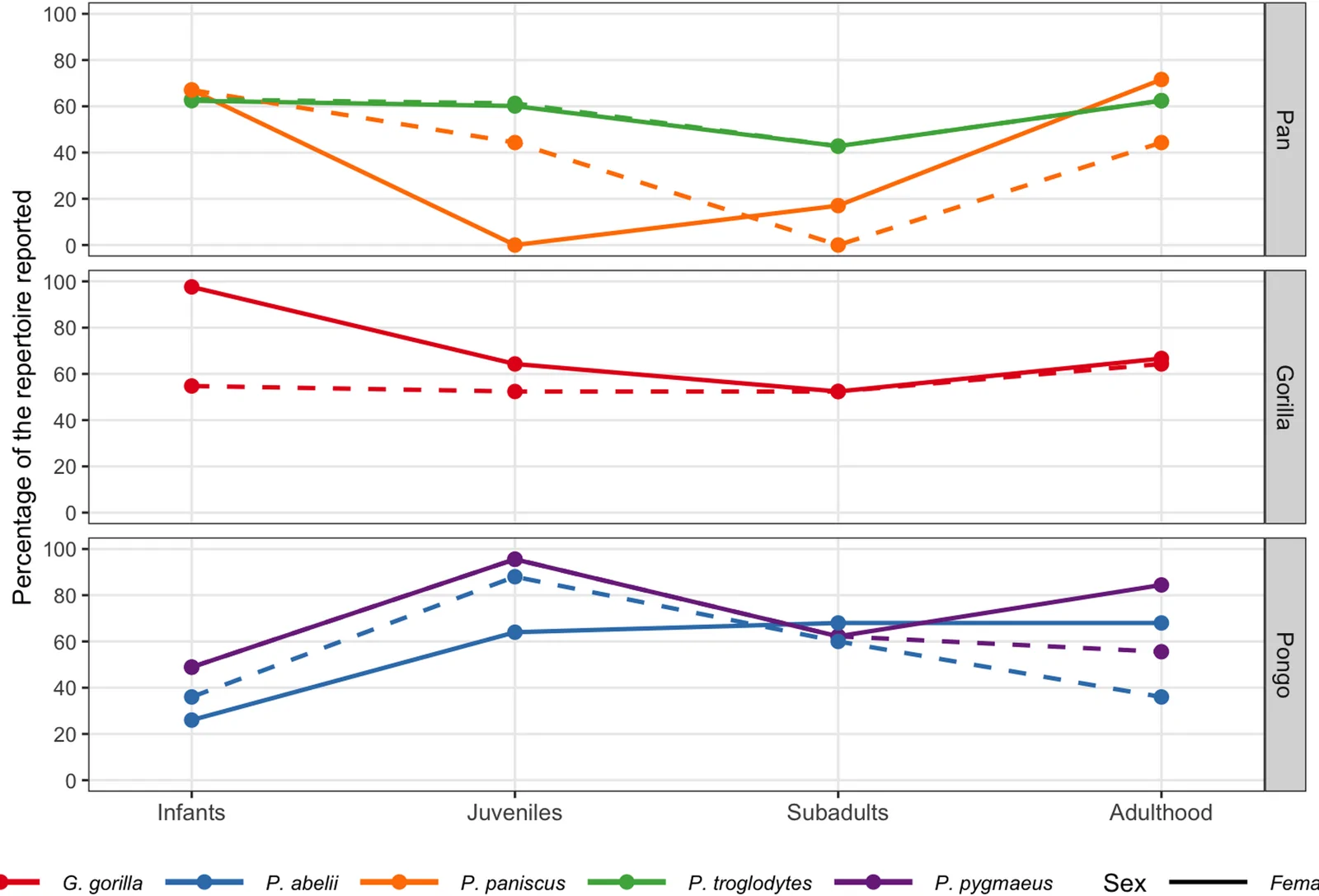
Proportion of play invitation signals identified across developmental stages for each species. Solid lines represent females, and dotted lines represent males. *Pan troglodytes* (green), *Pan paniscus* (orange), *Gorilla gorilla* (red), *Pongo pygmaeus* (purple), and *Pongo abelii* (blue)

When comparing across developmental stages, we observed that the largest proportion of play invitation signals occurred during early life stages, either infancy or juvenility, followed by a decrease during the subadult period (Fig. 4; Table 6). In females of most species, except in *P. abelii*, an additional increase was observed during adulthood.

In **chimpanzees**, play invitation signals were recorded in similar proportions across the lifespan for both sexes. A slight decrease was present in subadults of both sexes, with proportions remaining stable in adult males and comparable between adult males and females.

In **bonobos**, adult females exhibited a higher proportion of signals (71.6%) than adult males (44.3%). Only during the juvenile stage did males show a higher proportion (44.32%) than females, for whom no signals were documented (0.00%). This pattern later reverted in subadulthood, when a higher proportion of the repertoire was found (17.05%) in comparison to males (0.00%).

In **gorillas** (*G. gorilla*), the relative proportion of signals was higher in females than in males, except during the subadult stage, where a decline was observed in females, followed by higher proportion in adulthood.

In **Bornean orangutans** (*P. pygmaeus*), no major sex differences were detected, except during adulthood, when females showed a higher proportion (84.4%) than males (55.6%). The highest proportion across stages occurred during the juvenile period (96.0%).

In **Sumatran orangutans** (*P. abelii*), sex differences varied across stages. During infancy and juvenility, males exhibited a higher proportion of signals than females. From the subadult period onward, females showed a higher proportion of signals, a pattern that continued into adulthood. In males, the proportion declined sharply at the subadult stage, reaching values similar to those observed during infancy.

**Table 6:** Proportion (%) of play invitation signals observed at each developmental stage by species

Sex	Life stage			
	Infancy	Juvenility	Subadulthood	Adulthood
*P. troglodytes* (*n* = 173)				
Male	63.01	61.27	42.77	62.43
Female	62.43	60.12	42.77	62.43
*P. paniscus* (*n* = 88)				
Male	67.05	44.32	0.00	44.32
Female	67.05	0.00	17.05	71.59
*G. gorilla* (*n* = 42)				
Male	54.76	52.38	52.38	64.29
Female	97.62	64.29	52.38	66.67
*P. pygmaeus* (*n* = 45)				
Male	48.89	95.56	62.22	55.56
Female	48.89	95.56	62.22	84.44
*P. abelii* (*n* = 50)				
Male	36.00	88.00	60.00	36.00
Female	26.00	64.00	68.00	68.00

N represents the total number of unique play invitation signals identified in the repertoire of each species (i.e., the denominator from which stage- and sex-specific percentages were calculated). Values represent the percentage of the total signal repertoire identified for each species and sex

## Discussion

This systematic review examined phylogenetic and ontogenetic patterns in play invitation signals across great apes. Two main findings emerged. First, interspecific variation did not align consistently with expected phylogenetic relationships, suggesting that evolutionary proximity alone does not fully account for differences in communicative repertoires. Second, developmental trajectories showed a shared pattern across taxa: play invitation signals were most prevalent during early life stages, decreased during subadulthood, and increased again in adulthood—particularly among females.

### Phylogenetic patterns and implications for gesture origins

The phylogenetic comparison revealed a pattern of structural similarity that did not align with evolutionary history, and the MCA results bear on this question at two levels. At the first level, the primary axis of variation among signals was structured by the developmental stage and sex of signal producers rather than by species identity. Although the overall shape of developmental trajectories was broadly shared across taxa, the specific composition of the repertoire within each stage diverged between species in ways that did not map onto phylogenetic relatedness. At the second level, the spatial relationships among species-level centroids do not track phylogenetic relationships in any consistent way.

Hobaiter and Byrne (2011), building on the genetic codification hypothesis, attributed substantial overlap in gestural repertoires across great ape species to shared evolutionary inheritance, suggesting that more closely related taxa should show greater repertoire similarity. The data provide only partial support for this prediction. Sumatran and Bornean orangutans were among the closest pairs in the ordination space (centroid distance = 0.734), consistent with their shared evolutionary heritage. Beyond this pair, however, the pattern breaks down. The two *Pan* species were among the most distant in the ordination space (Pt–Pp = 3.302). *Gorilla gorilla*, meanwhile, was closer to both Pongo species (Gg–Pa = 0.645; Gg–Py = 1.060) than to either *Pan* species (Gg–Pp = 1.517; Gg–Pt = 3.349), even though gorillas are phylogenetically more closely related to *Pan* than to *Pongo*. Under a strictly phylogenetic account, the two *Pan* species would be expected to show the greatest mutual similarity; these quantitative patterns do not support that expectation.

As noted before, ontogenetic variation alone does not adjudicate between the two frameworks, since neither predicts the absence of developmental change in signal use. What the present findings do indicate is that the structure of interspecific similarity does not follow phylogenetic distance in any uniform way. This is more consistent with a model in which factors beyond evolutionary inheritance contribute to organising repertoire similarities across taxa, though identifying what those factors are would require data beyond what the present review can provide.

### Ontogenetic trajectories and developmental factors

Ontogenetic analyses revealed that infancy and juvenility represent key developmental windows for the use of play invitation signals. These stages consistently showed the highest repertoire diversity across species. The subsequent decline during the subadult period is consistent with reductions in play frequency documented for chimpanzees, bonobos, and orangutans during adolescence (Hayaki 1985; Mendoza-Granados and Sommer 1995; Pusey 1990; Kunz et al. 2024; van Adrichem et al. 2006), which limit opportunities for signal exchange. The increase observed in adult females across taxa may reflect shifts in social roles or interactional patterns later in life, likely higher interaction of mothers with offspring, previously documented in the literature (e.g., Schneider et al. 2012).

These developmental findings align with the hypothesis that play, especially during early life, may serve as a critical context for the acquisition of further skills. Empirical support for this link comes from wild chimpanzees, where social play has been associated with the attainment of key developmental milestones, including the onset of grooming with non-kin individuals, age at first independent travel, and first attempts to mate with females (Heintz et al. 2017). Comparable patterns have been documented outside the great apes: in Assam macaques, individuals engaging more frequently in play bouts develop locomotor abilities such as leaping at a faster rate (Berghänel et al. 2015). Whether such links extend specifically to communicative skill acquisition, including the development of play invitation repertoires, remains to be tested.

### Species-specific considerations and discrepancies with prior studies

#### Chimpanzees (Pan troglodytes)

Lonsdorf et al. (2014) documented a steady increase in the frequency of play invitation signals during the first five years of life. Our synthesis does not reproduce this pattern because we examined a different dimension of development: the presence of each signal type across ontogenetic stages, not its frequency of use. A signal may become more common with age without necessarily altering the structure or diversity of the repertoire—parameters central to our review. Moreover, aspects such as the particular combinations in which signals were sequenced, or increases in the elaboration of communicative displays over development — dimensions not examined in sufficient granularity within the present dataset — may further explain the developmental trends reported by Lonsdorf and colleagues.

Future analyses incorporating measures of complexity, persistence, and elaboration will be needed to clarify how these dimensions develop relative to repertoire composition.

#### Bonobos (Pan paniscus)

Bonobos exhibited a lower overall proportion of play invitation signals than chimpanzees. This lower proportion does not indicate a lower frequency of play signals but rather greater heterogeneity in repertoire composition across age classes. Two interpretations are possible. First, specific age categories in bonobo society may specialise in particular communicative displays, with infants using certain signal types that shift across later developmental stages. Second, the relatively small bonobo sample sizes in the reviewed studies, often focused on specific age groups, could artificially inflate stage-specific differences. Future work with larger datasets and controls for proximity, tolerance, and interaction rates would clarify the influence of social context.

#### Gorillas (Gorilla gorilla)

Patterns in gorillas showed moderate sex differences and stage-specific shifts, aligning partially with earlier findings on their play faces and rapid facial mimicry (Waller and Cherry 2012; Bresciani et al. 2022). However, the limited number of studies available for this genus constrains broader comparative inferences and highlights the need for expanded datasets.

#### Orangutans (Pongo spp.)

Juvenile orangutans exhibited the highest proportions of play invitation signals, consistent with developmental patterns in which immature individuals initiate more social contacts (Mitra Setia et al. 2008). Within the genus, the patterns observed for Bornean and Sumatran orangutans diverged from prior work. Fröhlich et al. (2022) reported more gesture repetitions in *P. abelii* than in *P. pygmaeus*. Repetition of gestures and repertoire breadth, however, refer to distinct dimensions: repetition concerns how often a signal is reused within an interaction, whereas repertoire breadth refers to the number of distinct signal types employed. Our analysis found higher repertoire proportions in *P. pygmaeus* across most developmental stages, indicating that the greater repetition documented in *P. abelii* does not preclude a broader repertoire of distinct play signals in *P. pygmaeus*.

These cross-species developmental patterns are consistent with a model in which the structure of variation in play invitation repertoires is shaped more strongly by socio-developmental factors than by phylogenetic relationships, supporting an account in which biological predispositions interact with social experience to shape communicative repertoires.

### Methodological considerations and limitations

Several methodological constraints must be acknowledged. First, differences in results across studies may stem from variation in how play invitation signals were defined, categorized, and coded. For example, Pollick and de Waal (2007) reported that bonobos rely more on limb-based signals, whereas this pattern did not emerge in our synthesis. Such discrepancies may reflect differences in morphological categories, communicative modalities, and ontogenetic periods used by different authors.

Second, our analyses relied on ethograms and datasets published by other researchers rather than on primary observations. This reliance limits control over sampling effort, observational context, and operational definitions, and may constrain the comparability of derived repertoires.

Third, studies varied substantially in sample sizes, group compositions, and environments (captive versus free-ranging), all of which may influence signal opportunities and thus the representation of the repertoire. Different sampling efforts across developmental stages could have led to differentiated conclusions drawn here. Repertoire-based analyses derived from other studies cannot control for variation in the number of individuals contributing to each age class, which may influence which signal types are documented for a given stage. Unlike analyses based on raw observational data, where subsampling procedures could be used to assess whether repertoire estimates approach asymptotic saturation, our study relies on presence-absence information, making such adjustments methodologically unfeasible. We note, however, that the MCA was conducted on signal-level structural variables rather than on repertoire counts per age class, and is therefore less directly susceptible to this form of sampling bias. Future systematic reviews addressing this question would benefit from restricting inclusion to studies that report asymptotically saturated individual repertoires.

An additional source of uncontrolled variation concerns factors related to social organisation that may shape the expression of play invitation signals but could not be incorporated into the present analysis. Differences in social structure, such as the male-bonded societies of chimpanzees compared to the female-bonded organisation of bonobos, may influence which signals are necessary to coordinate play and with which partners. The duration of established relationships within a group may similarly affect the frequency and diversity of signals used between particular dyads. Examining these factors requires individual-level data beyond the scope of the present review.

Finally, the current Pan-centrism in primate behavioral research (Lameira and Call 2020) likely yields stronger and more reliable inferences for Pan troglodytes than for other taxa, underscoring the need for more balanced research efforts across great ape species.

### Future directions

Several directions could meaningfully extend the findings reported here. A first priority concerns the granularity and consistency of signal coding: adopting standardized frameworks, such as that proposed by Grund et al. (2023), would allow future studies to capture dimensions beyond simple signal occurrence, including laterality, flexions, orientations, repetitions, and contact roles; and in doing so resolve much of the cross-study incomparability discussed above. Even with such frameworks in place, however, the uneven distribution of research effort across taxa would remain a limiting factor: bonobos and gorillas are represented by markedly fewer studies than chimpanzees and orangutans, and expanding and balancing sampling across species is a necessary complement to any coding improvement.

Beyond the cross-sectional repertoire comparisons conducted here, longitudinal datasets tracking the same individuals across developmental stages would allow researchers to test directly whether the ontogenetic patterns identified in this review reflect within-individual change rather than between-cohort variation, while also enabling the tracking of complexity, sequentiality, and multimodality as they develop over time. Such longitudinal data would, in turn, create the conditions needed to integrate social context variables —tolerance, proximity, and dominance relationships — that the present review could not control for, but which likely interact with developmental stages to shape signal use. None of these advances, finally, are achievable without greater investment in open data: making raw behavioural records available, rather than only published ethogram summaries, would allow future reviews to test these questions with the individual-level precision that comparative synthesis alone cannot provide.

The developmental variability observed across taxa suggests that great ape communication cannot be reduced to phylogenetic templates but rather reflects a dynamic interplay between social and ontogenetic factors.

## Data Availability

The source code and data used for generating all analyses are available in a GitHub repository. This information will be made available upon request to the corresponding author.
